# Notice of retraction: Auraptene-induced cytotoxicity mechanisms in human malignant glioblastoma (U87) cells: role of reactive oxygen species (ROS)

**DOI:** 10.17179/excli2024-7418

**Published:** 2024-05-23

**Authors:** Amir R. Afshari, Mohammad Jalili-Nik, Mohammad Soukhtanloo, Ahmad Ghorbani, Hamid R. Sadeghnia, Hamid Mollazadeh, Mostafa Karimi Roshan, Farzad Rahmani, Hamed Sabri, Mohammad Mahdi Vahedi, Seyed Hadi Mousavi

**Affiliations:** 1 Department of Physiology and Pharmacology, School of Medicine, North Khorasan University of Medical Sciences, Bojnurd, Iran; 2 Department of Pharmacology, Faculty of Medicine, Mashhad University of Medical Sciences, Mashhad, Iran; 3 Department of Clinical Biochemistry, Faculty of Medicine, Mashhad University of Medical Sciences, Mashhad, Iran; 4 Pharmacological Research Center of Medicinal Plants, Mashhad University of Medical Sciences, Mashhad, Iran; 5 Department of Pharmacology, School of Medicine, Zahedan University of Medical Sciences, Zahedan, Iran; 6 Health Promotion Research Center, Zahedan University of Medical Sciences, Zahedan, Iran; 7 Medical Toxicology Research Center, Mashhad University of Medical Sciences, Mashhad, Iran

## ⁯

This retraction concerns the article: Auraptene-induced cytotoxicity mechanisms in human malignant glioblastoma (U87) cells: role of reactive oxygen species (ROS) EXCLI Journal 2019;18:576-590. https://www.excli.de/index.php/excli/article/view/1404

The chief editor and the editorial board members of EXCLI J have retracted the following article:

Afshari AR, Jalili-Nik M, Soukhtanloo M, Ghorbani A, Sadeghnia HR, Mollazadeh H, Karimi Roshan M, Rahmani F, Sabri H, Vahedi MM, Mousavi SH. Auraptene-induced cyto-toxicity mechanisms in human malignant glioblastoma (U87) cells: role of reactive oxygen species (ROS). EXCLI J. 2019 Jul 30:18:576-590. doi: 10.17179/excli2019-1136. eCollection 2019. Available from: https://www.excli.de/index.php/excli/article/view/1404

After the article was published, concerns emerged that the same authors had reported similar findings in the following publication.

Afshari AR, Karimi Roshan M, Soukhtanloo M, Ghorbani A, Rahmani F, Jalili-nik M, Vahedi MM, Hoseini A, Sadeghnia HR, Mollazadeh H, Mousavi SH. Cytotoxic effects of auraptene against a human malignant glioblastoma cell line. Avicenna J Phytomed. 2019 Jul-Aug;9(4):334-346. https://pubmed.ncbi.nlm.nih.gov/31309072/

The journal noticed that both articles described very similar results leading to the same conclusions (apoptotic effects of auraptene). It was also noticed that some conclusions have been made based on data from only two separate experiments (Western blot experiments). It was found that the two articles were reviewed and published at the same time without in-forming the editorial office of EXCLI J of the submission to Avicenna J Phytomed.

The corresponding author has been contacted but did not provide a sufficient explanation to our queries. Therefore, we cannot guarantee this article's reliability or integrity. According to the reply, the authors disagree with our decision. The chief editor and the editorial board members made this decision taking into consideration our editorial policies and the COPE guidelines (https://publicationethics.org/guidance/Guideline according to the best of our knowledge and belief. The retracted article will be available online but marked as “Retracted”.

